# Diagnostic Fragment-Ion-Based for Rapid Identification of Chlorogenic Acids Derivatives in *Inula cappa* Using UHPLC-Q-Exactive Orbitrap Mass Spectrometry

**DOI:** 10.1155/2021/6393246

**Published:** 2021-08-20

**Authors:** Jie Peng, Jing Xie, Silin Shi, Lilan Luo, Kailin Li, Pei Xiong, Wei Cai

**Affiliations:** ^1^School of Pharmaceutical Sciences, Hunan Province Key Laboratory for Antibody-Based Drug and Intelligent Delivery System, Hunan University of Medicine, Huaihua 418000, China; ^2^Department of Rehabilitation Medicine and Health Care of Hunan Medical College, Hunan University of Medicine, Huaihua 418000, China; ^3^Hunan Provincial Key Laboratory of Dong Medicine, Hunan University of Medicine, Huaihua 418000, China

## Abstract

*Inula cappa* (Buch.-Ham. ex D. Don) DC has been used in traditional Chinese medicine to treat malaria, dysentery, and hepatitis. Previous studies have shown that chlorogenic acid is the effective ingredient of plants in this family. And the research of the chlorogenic acid in *Inula cappa* will help to further improve the effective resource utilization rate of this plant. Therefore, it is necessary to establish an accurate method to characterize the chlorogenic acid components in *Inula cappa*. In this study, a simple, fast, and sensitive UHPLC-Q-Exactive Orbitrap mass spectrometry method was established, which can simultaneously analyze known and unknown ingredients in a short time (within 30 minutes) in *Inula cappa.* According to the diagnosis fragmentation ions, retention time, and bibliography, 68 chlorogenic acid derivatives were identified in *Inula cappa*. The results of this experiment lay the foundation for the active substances and quality control of *Inula cappa* and provide a theoretical basis for whether *Inula cappa* can be an alternative to the endangered wild medicinal materials of the same family.

## 1. Introduction

Traditional Chinese medicine (TCM) has been applied for thousands of years in China and its surrounding areas. For a long time, TCM has attracted worldwide attention because of its extremely effective treatment of certain diseases and minimal side effects. It was estimated that more than 1.5 billion people all over the world trust in the efficacy of TCM and rest assured of its safety [1]. However, it is still unclear which of the ingredients of TCM are effective for the treatment of diseases because they are a complex mixture of hundreds of different chemical components. It not only hinders the clinical promotion and application of TCM but also greatly hinders the development of TCM. In recent years, with the continuous improvement and advancement of analytical techniques and methodology, the research speed of TCM has been greatly accelerated.

Based on the diversity and complexity of the chemical components of TCM, it is necessary to use advanced analytical techniques and methods to explain the pharmacological basis and mechanism of action of TCM because ultrahigh performance liquid chromatography-mass spectrometry (UHPLC-MS) with high sensitivity is to reduce sample complexity [2]. In addition, UHPLC-MS also has the advantages of good specificity, short analysis cycle, and good signal reproducibility [3, 4]. UHPLC-HRMS has now been widely used in the analysis and identification of TCM components, especially most small molecules.

*Inula cappa* (Buch-Ham. ex D. Don) DC, a perennial shrub, belonging to the family composite, known as “Yang Er Ju” in Chinese, is widely distributed in the southern areas of China, such as Hunan, Sichuan, Yunnan, Guizhou, Guangxi, Guangdong, and Zhejiang provinces. Its roots or the whole plant have traditionally been used as medicines for treatment of malaria, dysentery, and hepatitis [4]. The compounds responsible for the various pharmacological effects of *Inula cappa* consist of sesquiterpene lactones (isoalantolactone and germacranolide), triterpenoids (lupeol, oleanolic acid, and *β*-sitosterol), steroids, anthraquinones, flavonoids (luteolin, apigenin, and chrysoeriol), fragrances, amides, and chlorogenic acid [5, 6].

Chlorogenic acids (CGAs) are the family of esters phytochemicals formed between cinnamic acid derivatives and (−)-quinic acids. In recent years, chlorogenic acid have been proven to mediate for its hepatoprotective, choleretic, antimicrobial activities, antioxidant, hypoglycaemic, and antiviral activities; besides, basic and clinical investigations have implied that the consumption of chlorogenic acid can reduce the relative risks of type 2 diabetes, obesity, and Alzheimer's disease [7, 8].

In this study, a simple, fast, and sensitive UHPLC-Q-Exactive Orbitrap mass spectrometry method was established, based on accurate mass, which can simultaneously analyze the known and unknown components in *Inula cappa* in a short time (within 30 minutes).

First, extracting the CGAs from the *Inula cappa* by refluxing, a high-resolution mass spectrum was obtained by using UHPLC-Q-Exactive Orbitrap MS in the negative mode and parallel reaction monitoring. Finally, the diagnostic fragment ions, retention time, and bibliography were established to process the ion chromatograms, and 68 compounds in the *Inula cappa* were rapidly identified.

## 2. Materials and Methods

### 2.1. Chemicals and Reagents

HPLC grade of methanol, acetonitrile, and formic acid were purchased from Fisher scientific Company (New jersey, USA). Standards substances of the following phytochemicals were purchased from Chengdu Herbpurify Co., Ltd. (Chengdu, China): trans-3-caffeoylquinic acid (neochlorogenic acid, X-014-170309), trans-4-caffeoylquinic acid (cryptochlorogenic acid, Y-067-180320), trans-5-caffeoylquinic acid (chlorogenic acid, L-007-171216), 3,5-dicaffeoylquinic acid (isochlorogenic acid A, Y-068-170903), 3,4-dicaffeoylquinic acid (isochlorogenic acid B, Y-069-180105), 4,5-dicaffeoylquinic acid (isochlorogenic acid C, Y-070-170515), 1,3-dicaffeo-pyridinequinic acid (cimarin, MUST-16022610), and 1,5-dicaffeo-pyridinequinic acid (cimarin, MUST-16022610).

### 2.2. Materials

The decoction pieces of *Inula cappa* (dried root) were purchased from Bozhou Jianzheng Trading Co., Ltd., (Henan, China), which were grounded into powder before sample preparation. The voucher specimen was deposited at School of Pharmaceutical Sciences, Hunan University of Medicine.

### 2.3. Instrumentation

A Q-Exactive Focus Orbitrap MS (Thermo Electron, Bremen, Germany) was connected to the Thermo Scientific Dionex Ultimate 3000 RS (Thermo Fisher Scientific, California, USA) via an ESI source. A UPW-N Series Water Purification System was purchased from LeiCi, Shanghai Yidian Scientific Instrument Co., Ltd., Shanghai, China. An automatic dual range professional type of analytical balances PTX-FA210 was purchased from Huazhi Electronic Technology Co., Ltd., Fujian, China.

### 2.4. Preparation of Standard Solutions

Accurately weigh 10 mg of each reference standard, and dissolve it in 10 mL of methanol; then, take 10 *μ*L and dilute to 1 mL. A volume of 1 *μ*L was injected into UHPLC-Q-Exactive Orbitrap MS for analysis.

### 2.5. Preparation of Sample Solutions

The dried powder of *Inula cappa* (10 g) was reflux-extracted in 50 mL 70% aqueous ethanol for 1 h, and then, the extracted solution was filtered and dried by rotary evaporation and then reconstituted with 10 ml methanol. A volume of 2 *μ*L was injected into UHPLC-Q-Exactive Orbitrap MS for analysis.

### 2.6. HPLC Chromatographic Condition

An Hypersil GOLD aQ (100 mm × 2.1 mm, 1.9 *μ*m) was used for chromatographic separation at 35°C. The mobile phase consisted of 0.1% formic acid (A) and acetonitrile (B) at a flow rate of 0.3 mL/min in the following gradient: 0 min, 5% B; 2 min, 10% B; 5 min, 20% B; 10 min, 25% B; 12 min, 55% B; 20 min, 80% B; 25 min, 95% B; 26 min, 5% B; and 30 min, 5% B.

All samples were analyzed in the negative mode as the following tune method. The nitrogen (purity ≥ 99.99%) served as sheath gas and auxiliary gas at the flow rate of 30 and 10 (arbitrary unit), respectively; the capillary temperature is 320°C; the auxiliary gas heater temperature is 350°C; and spray voltage is 3.5 kV. High-resolution mass spectrum was acquired at full scan in a mass range of m/z 120–1000 at a resolution of 35000 detected by the Orbitrap analyzer. The MS^2^ data at a resolution of 17500 were obtained by the parallel reaction monitoring mode triggered by the inclusion ions list. The nitrogen (purity ≥ 99.999%) served as collision gas to generate the fragment ions, and the energy was set as normalized collision energy 30% [9].

### 2.7. The Establishment of Diagnosis Fragmentation Ions

Chlorogenic acid (CGA) is a series of ester phytochemicals formed between caffeic acid and quinic acid. It is easy to understand that all CGAs use quinic acid as the backbone and produce similar fragments, which can be defined as diagnostic ions. By using diagnostic ion information, CGAs can be quickly screened and characterized. The fragmentations behavior of CGAs has been reported and summarized in the previous literature.

For example, for CGAs with the same quinic acid as the backbone of quinine, cinnamoyl residues (such as caffeoyl and ferulic acid esters) are usually cleaved and lost on the ester bond, thus yielding about 191.0552 at m/z, and the characteristic product ion corresponds to [quinic acid-H]-(C_7_H_12_O_6_). The diagnostic ions at m/z 353.0875 and m/z 515.1195 correspond to mono-CQA and diCQA m/z 191.0561 and m/z 173.0455 are present in all types of chlorogenic acid and can be used as diagnostic ions. The ions with mass numbers m/z 173 and m/z 193 derived from dehydroquinic acid and ferulic acid are used as diagnostic ions, which can diagnose FQA (especially 4FQA) and CQA at the same time [10].

### 2.8. Data Processing and Analysis

LC-MS data analysis was performed using Xcalibur software version 4 (Thermo Fisher Scientific, San Jose, California, USA). The raw data including the full-scan MS and MS^2^ data were processed by the Compound Discover 3.0 using the expected compounds predicted method [11] based on the metabolism workflow templates to detect the chlorogenic acid derivatives constituents of *Inula cappa*. Finally, candidates for CGA were characterized based on the diagnostic fragment ions, retention time, and bibliography.

## 3. Result

**Figure 1:**
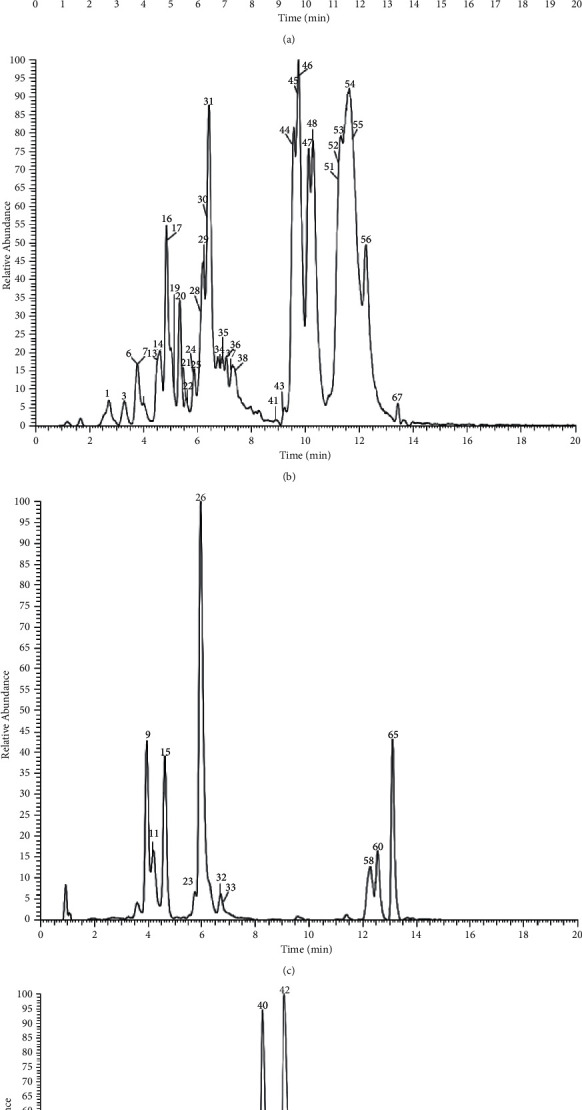
The high-resolution extracted ion chromatogram (HREIC) in 5 ppm for the multiple compounds in *Inula cappa*. (a) 353.10894, 529.15628, 661.15628, and 721.17741; (b) 335.07724, 367.10346, 497.10893, 499.12458, 499.14571, 515.14063, 529.13515, 677.17232, and 839.22515; (c) 337.09289, 341.08781, and 691.16684; (d) 353.08781, 515.1195, and 677.15119.

The table lists all the chlorogenic acid and its derivatives detected in the extracted *Inula cappa* sample by UHPLC-Q-Exactive Orbitrap mass spectrometry based on diagnostic fragment ions, retention time, and bibliographical identification (Table 1). A total of 68 chlorogenic acids and their derivatives were identified (Figure 1).

### 3.1. Identification of Monoacyl-Quinic Acids or Monoacyl-Shikimic Acids

Compounds 14, 19, 22, 24, 28, 30, 34, and 41 generated the same quasimolecular ions [M-H]^−^ at m/z 335.076 (C_16_H_15_O_8_) which eluted at 4.63, 5.15, 5.65, 5.81, 6.14, 6.38, 6.86, and 8.32 min, respectively. According to previously reported literature, these compounds may be either caffeoylquinic acid lactones (CQL) or caffeoylshikimic acids (CSA). Due to the loss of lactone and H_2_O moiety, quinic acid lactones prone to generate ions at m/z 161.023, so m/z 161.023 can be used to distinguish CQLs and CSAs. For the above reasons, these compounds were tentatively identified as 3-CQL, 1-CQL, 4-CQL, 4-CSA, 3-CSA, 1-CQL, 4-CQL, and 1-CQL, respectively [12–15].

Compounds 23, 26, and 32 were eluted at 5.76, 5.97, and 6.71 min and showed a deprotonated molecular ion [M-H]^−^ at m/z 337.09299 (1.20 ppm, C_16_H_17_O_8_), 337.09320 (1.41 ppm, C_16_H_17_O_8_), and 337.09351 (1.71 ppm, C_16_H_17_O_8_), respectively. According to previous literature analysis, the base peak ions and fragment ions of the MS^2^ spectrum were temporarily designated as 5-O-p-coumaroylquinic acid (pCoQA), 5-pCoQA, and 1-pCoQA [16].

Compound 6 was eluted at 3.76 min, with the deprotonated ion [M-H]^−^ at m/z 499.14609 (1.47 ppm, C_22_H_27_O_13_), 162 Da (C_6_H_10_O_5_) more than pCoQA (C_16_H_17_O_8_). The fragment ion generated at m/z 337.09 (C_16_H_17_O_8_) by loss the C_6_H_10_O_5_ moiety was detected in MS^2^ spectrum of compound 6, indicating that it was hexoside of pCoQA. The fragment ions at m/z 173.044 and 191.054 were shown in the MS^2^ spectrum of compound 6, indicating that compound 6 was 4-pCo, 5CQA, respectively [12–14].

Compounds 2, 4, and 18 were eluted at 2.85, 3.33, and 4.89 min and generated the same deprotonated ion [M-H]^−^ at m/z 353.08 (C_16_H_17_O_9_). According to comparing the retention time, MS data with those reference standards, compounds 2, 4, and 18 were accurately characterized as cis-3-CQA, trans-3-CQA, and trans-5-CQA [17].

Compounds 1, 3, 7, 10, and 13 eluted at 2.71, 3.28, 3.76, 4.00, and 4.50 min and showed a deprotonated molecular ion [M-H]^−^ at m/z 515.14050 (−0.25 ppm, C_22_H_27_O_14_), 515.13959 (−2.02 ppm, C_22_H_27_O_14_), 515.14069 (0.12 ppm, C_22_H_27_O_14_), 515.14081 (0.35 ppm, C_22_H_27_O_14_), and 515.14105 (0.82 ppm, C_22_H_27_O_14_), 162 Da (C_6_H_10_O_5_) more than CQA, indicating they were the hexoside of CQA. The presence of fragment ion at m/z 323.076 (C_15_H_15_O_8_) can distinguish the attachment sites of hexosides. In summary, 1, 3, and 7 are tentatively designated as CQA-4′-hexoside, and the others may be CQA-3′-hexoside [12, 14, 18].

Compounds 16, 29, 31, and 53 were eluted at 4.85, 6.23, 6.42, and 11.30 min and generated deprotonated ions [M-H]^−^ at m/z 367.10333 (0.97 ppm, C_17_H_19_O_9_), 367.10352 (1.16 ppm, C_17_H_19_O_9_), 367.10352 (1.16 ppm, C_17_H_19_O_9_), and 367.10199 (−3.99 ppm, C_17_H_19_O_9_), respectively, indicating they were the feruloylquinic acid (FQA). According to the base peak ion generated at m/z 193.049 (C_10_H_9_O_4_), it was determined to be 3-FQA. The base peak ion generated at m/z 173.044 (C_7_H_9_O_5_) was determined to be 4FQA; the base peak ion generated at m/z 191.054 (C_7_H_11_O_6_) was determined to be 5FQA. Above all, they were tentatively characterized as 3FQA, 4FQA, 5FQA, and 4FQA [16, 17].

Compounds 5 and 12 with the deprotonated ions [M-H]^−^ at m/z 529.15628 (−2.02 ppm, C_23_H_29_O_14_) and 529.15649 (0.40 ppm, C_23_H_29_O_14_) were eluted at 3.33 and 4.39 min, 162 Da (C_6_H_10_O_5_) more than FQA, indicating they were the hexoside of FQA. The presence of fragment ions m/z 193.049, 173.044, and 367.102 further confirmed the above identification. The base peak ion of m/z 529 can be used to distinguish the submitted position of the above groups. In summary, compound 5 was tentatively identified as 3-FQA-hexosides, and 12 is 4FQA-hexosides [12–14].

### 3.2. Identification of Diacyl-Quinic Acids or Diacyl-Shikimic Acids

Compounds 51, 55, and 56 possessed deprotonated ions [M-H]^−^ at m/z 497.10956 (1.72 ppm, C_25_H_21_O_11_), 497.10944 (1.60 ppm, C_25_H_21_O_11_), and 497.10950 (1,66 ppm, C_25_H_21_O_11_) and were eluted at 11.20, 11.68, and 12.24 min, which could be dicaffeoylquinic acid lactones (DiCQL) or dicaffeoylshikimic acids (DiCSA). Compounds 51, 55, and 56 had the same base peak at m/z 161.0230 by the loss of lactone and H_2_O part of quinine lactone, so they were tentatively named DICQL [12–14].

Compounds 36, 37, 43, 45, 52, and 67 were eluted at 7.05, 7.23, 9.22, 9.72, 11.26, and 13.41 min and showed the deprotonated ion [M-H]^−^ at m/z 499.12653 (3.89 ppm, C_25_H_23_O_11_), 499.12396 (0.47 ppm, C_25_H_23_O_11_), 499.12460 (1.11 ppm, C_25_H_23_O_11_), 499.12515 (1.66 ppm, C_25_H_23_O_11_), 499.12451 (1.02 ppm, C_25_H_23_O_11_), and 499.12097 (−2.52 ppm, C_25_H_23_O_11_), respectively. In the MS^2^ spectra of these compounds, the characteristic fragment ions m/z 173.044 (C_7_H_9_O_5_), 179.033 (C_9_H_7_O_4_), and 191.054 (C_7_H_11_O_6_) of p-coumaroyl-caffeoylquinic acids (pCoCQA) were appeared, indicating that they were pCoCQA. According the base peak and retention time, the absence of base peak at m/z 173.044 (C_7_H_9_O_5_) of compounds 36, 37, 43, 45, and 67 are consistent with their being 3C, 5pCoQA, Cis-3-pCo, 5CQA, Cis-3-pCo, 5CQA, 3C, 5-pCoQA, and 3C, 5-pCoQA. Besides, compound 52 was tentatively identified as 4C, 5-pCoQA [15, 19].

Compounds 27, 39, 40, and 42 were eluted at 6.08, 8.02, 8.31, and 9.12 min and had the same quasimolecular ions [M-H]^−^ at m/z 515.119 (C_25_ H_23_ O_12_), possessed the same retention time and mass spectrum data with these reference standards 1,3-diCQA, 3,4-diCQA, 3,5-diCQA, and 4,5-diCQA, respectively. Thus, they were unambiguously assigned as 1,3-diCQA, 3,4-diCQA, 3,5-diCQA, and 4,5-diCQA [20, 21].

Compounds 21, 25, and 38 with the deprotonated ion [M-H]^-−^at m/z 677.17285 (0.78 ppm, C_31_H_33_O_17_), 677.17468 (3.48 ppm, C_31_H_33_O_17_), and 677.17145 (−1.30 ppm, C_31_H_33_O_17_), respectively, were eluted at 5.56, 5.88, and 7.38 min, 162 Da (C_6_H_10_O_5_) more than diCQA, suggesting they were the hexoside of diCQA, which were further confirmed by the presence of fragment ions m/z 515.117 (C_25_H_23_O_12_), 173.044 (C_7_H_9_O_5_), 179.033 (C_9_H_7_O_4_), and 191.054 (C_7_H_11_O_6_). Therefore, they were inferred as diCQA-hexoside [12–14, 17].

Compounds 17 and 20 were eluted at 4.87 and 5.34 min, yielded the same deprotonated ion [M-H]^−^ at m/z 839.225 (−3.62 ppm, C_37_H_43_O_22_), 162 Da (C_6_H_10_O_5_) more than diCQA-hexoside, suggesting they were the dihexoside of diCQA. Therefore, they were tentatively identified as diCQA-dihexoside.

Compounds 35, 44, 46, 47, 48, and 54 were eluted at 6.93, 9.54, 9.76, 10.11, 10.28, and 11.62 min and generated a quasimolecular ion [M-H]^−^ at m/z 529.13611 (1.82 ppm, C_26_H_25_O_12_), 529.13525 (0.19 ppm, C_26_H_25_O_12_), 529.13550 (0.66 ppm, C_26_H_25_O_12_), 529.13550 (0.66 ppm, C_26_H_25_O_12_), 529.13550 (0.66 ppm, C_26_H_25_O_12_), and 529.13525 (0.19 ppm, C_26_H_25_O_12_). These fragment ions at m/z 173.044 (C_7_H_9_O_5_), 193.049 (C_10_H_9_O_4_) or 353.086 (C_16_H_17_O_9_), and 367 (C_17_H_19_O_9_) were shown in all of those above compounds, which were consistent with caffeoylferuloylquinic acids (CFQA). Based on the retention time, data of MS^2^, and diagnosis ions in bibliography, compounds 35, 44, 46, 47, 48, and 54 were tentatively identified as 3C, 5FQA, 3F, 5CQA, 3F, 5CQA, 3F, 5CQA, 3C, 5FQA, and 4C, 5FQA, respectively [16, 20].

### 3.3. Identification of Triacyl-Quinic Acids or Triacyl-Shikimic Acids

Compounds 57 and 59 yielded a deprotonated ion [M-H]^−^ at m/z 661.15472 (−2.36 ppm, C_34_H_29_O_14_) and 661.15369 (−3.92 ppm, C_34_H_29_O_14_) and were eluted at 12.27 and 12.38 min, respectively. Based on the reference, retention time, fragment ions m/z 173.044 (C_7_H_9_O_5_), 179.033 (C_9_H_7_O_4_), 337.092 (C_16_H_17_O_8_), and 353.087 (C_16_H_17_O_9_) indicated that they are p-coumaroyl-dicaffeoylquinic acids (pCoDiCQA).

Compounds 49, 50, and 62 were eluted at 10.32, 10.75, and 12.72 min, generated a quasimolecular ion [M-H]^−^ at m/z 677.15173 (0.75 ppm, C_34_H_29_O_15_), 677.15179 (0.88 ppm, C_34_H_29_O_15_), and 677.15173 (0.79 ppm, C_34_H_29_O_15_), respectively. Based on the existence of fragment ions m/z 353.0869 (C_16_H_17_O_9_) and m/z 515.1180 (C_25_H_23_O_12_), the absence of ion m/z 497.1070 (C_25_H_21_O_11_) of compounds 49, 50, and 62 were tentatively identified as 1,3-C, 1,3,4-TriCQA and 1,4,5-TriCQA [20].

Compounds 58, 60, and 65 were eluted at 12.28, 12.56, and 13.11 min, with a quasimolecular ion [M-H]^−^ at m/z 691.16840 (2.25 ppm, C_35_H_31_O_15_), 691.16821(1.98 ppm, C_35_H_31_O_15_), and 691.16766 (1.18 ppm, C_35_H_31_O_15_), respectively. The fragment ions m/z 529.133 (C_26_H_25_O_12_), 367.118 (C_21_H_19_O_6_), 173.044(C_7_H_9_O_5_), and 179.033 (C_9_H_7_O_4_) were detected in MS^2^ data of those compounds, indicating that they were dicaffeoylferuloylquinic acids (DiCFQA).

According to the above principles, compounds 61, 63, 64, 66, and 68 can be identified as dicaffeoyl-sinapoylquinic acid (DiCSQA).

### 3.4. Others

Compounds 9, 11, 15, and 33 were eluted at 3.97, 4.16, 4.64, and 6.73 min and yielded a deprotonated ion [M-H]^−^ at m/z 341.08798 (1.27 ppm, C_15_H_17_O_9_), 341.08801 (1.30 ppm, C_15_H_17_O_9_), 341.08801 (1.30 ppm, C_15_H_17_O_9_), and 341.08786 (0.16 ppm, C_15_H_17_O_9_), which show a fragment ion at m/z 179.033 (C_9_H_7_O_4_) by losing the 162 Da in the MS^2^ experiment. The fragment ions of m/z 135.043 (C_8_H_7_O_2_) and 179.033 (C_9_H_7_O_4_) consisted with the caffeic acids; above all, they were tentatively identified as caffeoyl hexoside (CA-hexoside) [22].

Compound 8 was detected at 3.83 min and generated the quasimolecular ion [M-H]^−^ at m/z 353.10687 (−0.97 ppm, C_13_H_21_O_11_). This compound yielded fragment ions at m/z 191.055 (C_7_H_11_O_6_) and 173.044 (C_7_H_9_O_5_), and guess was composed of quinic acid fragments. Therefore, it might be considered as hexoside of quinic acid (QA-hexoside) [23].

## 4. Conclusions

In this study, 68 chlorogenic acid and derivatives exhaustively characterized from the extract of *Inula cappa* by using the strategy for the rapid detection and identification of CGAs using UHPLC-Q-Exactive Orbitrap mass spectrometry with diagnostic fragment ion technology was proposed. The developed strategy was proved reliable and efficient in rapid discovery of new CGA class from *Inula cappa*. The results will provide new insights into the effective substances and quality control of *Inula cappa*. At the same time, the yield of *Inula cappa* is very high, and the study of the chlorogenic acid can be used as a basis to judge whether it can be used as a substitute for endangered wild medicinal materials. Besides, it also provides new insights for understanding the qualitative characteristics of these phytochemical components in other TCM.

## Figures and Tables

**Table 1:** The retention time and mass spectrometric data of CGAs in *Inula cappa*.

Peak	*t*_*R*_	Theoretical mass (m/z)	Experimental mass (m/z)	Error (ppm)	Formula [M-H]	MS/MS fragment	Identification/reactions
1	2.71	515.14063	515.14050	−0.25	C_22_H_27_O_14_	MS^2^ [515]: 179.0343 (100), 191.0555 (21)	CQA-4′-hexoside
2	2.85	353.08781	353.08798	1.27	C_16_H_17_O_9_	MS^2^ [353]: 191.0554 (100)	Cis-3-CQA
3	3.28	515.14063	515.13959	−2.02	C_22_H_27_O_14_	MS^2^ [515]: 179.0342 (100), 191.0554 (27), 341.0878 (16)	CQA-4′-hexoside
4	3.33	353.08781	353.08789	1.18	C_16_H_17_O_9_	MS^2^ [353]: 191.0554 (100), 135.0440 (82), 179.0341 (45)	Trans-3-CQA
5	3.33	529.15628	529.15521	−2.02	C_23_H_29_O_14_	MS^2^ [529]: 193.0499 (100), 191.0554 (35), 173.0447 (25)	3-FQA-hexoside
6	3.76	499.14571	499.14609	1.47	C_22_H_27_O_13_	MS^2^ [499]: 173.0447 (100), 93.0333 (80), 191.0555 (24), 163.0392 (14)	4-pCo, 5CQA
7	3.76	515.14063	515.14069	0.12	C_22_H_27_O_14_	MS^2^ [515]: 191.0555 (100)	CQA-4′-hexoside
8	3.83	353.10894	353.10687	−0.97	C_13_H_21_O_11_	MS^2^ [353]: 191.0554 (100), 85.02814 (7), 173.0449 (4)	QA-hexoside
9	3.97	341.08781	341.08798	1.27	C_15_H_17_O_9_	MS^2^ [341]: 135.0441 (100), 179.0342 (59)	CA-hexoside
10	4.00	515.14063	515.14081	0.35	C_22_H_27_O_14_	MS^2^ [515]: 173.0447 (100), 179.0342 (62), 191.0554 (43), 353.0884 (3)	CQA-3′-hexoside
11	4.16	341.08781	341.08801	1.30	C_15_H_17_O_9_	MS^2^ [341]: 135.0441 (100), 179.0342 (59), 161.0235 (14)	CA-hexoside
12	4.39	529.15628	529.15649	0.40	C_23_H_29_O_14_	MS^2^ [529]: 173.0447 (100), 191.0554 (25), 111.0439 (20), 193.0499 (13)	4FQA-hexoside
13	4.50	515.14063	515.14105	0.82	C_22_H_27_O_14_	MS^2^ [515]: 191.0554 (100), 161.0235 (14)	CQA-3′-hexoside
14	4.63	335.07724	335.07733	1.19	C_16_H_15_O_8_	MS^2^ [335]: 161.0235 (100), 173.0600 (22)	3-CQL
15	4.64	341.08781	341.08801	1.30	C_15_H_17_O_9_	MS^2^ [341]: 135.0441 (100), 179.0342 (71), 161.0235 (8)	CA-hexoside
16	4.85	367.10346	367.10333	0.97	C_17_H_19_O_9_	MS^2^ [367]: 134.0362 (100), 193.0499 (59)	3-FQA
17	4.87	839.22515	839.22515	0.23	C_37_H_43_O_22_	MS^2^ [839]: 353.0881 (100), 191.0556 (76), 173.0448 (8)	DiCQA-dihexoside
18	4.89	353.08781	353.08783	1.12	C_16_H_17_O_9_	MS^2^ [353]: 191.0554 (100), 135.0441 (20), 173.0447 (15), 179.0342 (9)	Trans-5-CQA
19	5.15	335.07724	335.07678	−1.37	C_16_H_15_O_8_	MS^2^ [335]: 161.0235 (100), 201.0553 (55), 59.0125 (52), 173.0816 (24)	1-CQL
20	5.34	839.22515	839.22595	0.96	C_37_H_43_O_22_	MS^2^ [839]: 341.0878 (100), 515.1416 (76), 179.0342 (32), 335.0775 (16), 353.0884 (5)	DiCQA-dihexoside
21	5.56	677.17232	677.17285	0.78	C_31_H_33_O_17_	MS^2^ [677]: 515.1409 (100), 341.0879 (87), 179.0342 (64), 353.0879 (32), 191.0553 (18)	DiCQA-hexoside
22	5.65	335.07724	335.07730	1.16	C_16_H_15_O_8_	MS^2^ [335]: 161.0235 (100), 135.0440 (55), 179.0339 (15), 173.04453 (7)	4-CQL
23	5.76	337.09289	337.09299	1.20	C_16_H_17_O_8_	MS^2^ [337]: 173.0447 (100), 163.0392 (25)	5-pCoQA
24	5.81	335.07724	335.07785	1.71	C_16_H_15_O_8_	MS^2^ [335]: 135.0441 (100), 179.0343 (31)	4-CSA
25	5.88	677.17232	677.17468	3.48	C_31_H_33_O_17_	MS^2^ [677]: 353.0880 (100), 179.0342 (92), 191.0555 (48), 341.0878 (30), 335.0775 (25), 173.0447 (10)	DiCQA-hexoside
26	5.97	337.09289	337.09320	1.41	C_16_H_17_O_8_	MS^2^ [337]: 191.0554 (100), 163.0392 (15)	5-pCoQA
27	6.08	515.11950	515.11969	1.29	C_25_H_23_O_12_	MS^2^ [515]: 191.0554 (100), 179.0342 (95), 135.0441 (14), 353.0880 (14)	1,3-DiCQA
28	6.14	335.07724	335.07755	1.41	C_16_H_15_O_8_	MS^2^ [335]: 135.0441 (100), 161.0235 (97), 173.0447 (51), 179.0342 (37)	3-CSA
29	6.23	367.10346	367.10352	1.16	C_17_H_19_O_9_	MS^2^ [367]: 173.0447 (100), 93.0333 (33), 134.0362 (32), 193.0500 (13)	4FQA
30	6.38	335.07724	335.07727	1.13	C_16_H_15_O_8_	MS^2^ [335]: 161.0235 (100), 135.0441 (25)	1-CQL
31	6.42	367.10346	367.10352	1.16	C_17_H_19_O_9_	MS^2^ [367]: 191.0554 (100), 93.0333 (54), 134.0362 (19), 173.0447 (17), 193.0500 (13)	5FQA
32	6.71	337.09289	337.09351	1.71	C_16_H_17_O_8_	MS^2^ [337]: 191.0555 (100), 173.0599 (72), 163.0392 (28), 119.0491 (19)	1-pCoQA
33	6.73	341.08781	341.08786	0.16	C_15_H_17_O_9_	MS^2^ [341]:135.0441 (100), 179.0343 (18), 161.0599 (16)	CA-hexoside
34	6.86	335.07724	335.07727	1.13	C_16_H_15_O_8_	MS^2^ [335]: 161.0235 (100), 173.1174 (12), 135.0040 (11)	4-CQL
35	6.93	529.13515	529.13611	1.82	C_26_H_25_O_12_	MS^2^ [335]: 179.0342 (100), 191.0554 (91), 205.0324 (30)	3C, 5FQA
36	7.05	499.12458	499.12653	3.89	C_25_H_23_O_11_	MS^2^ [335]: 179.0342 (100), 191.0554 (91), 205.0324 (30)	3C, 5-pCoQA
37	7.23	499.12458	499.12396	0.47	C_25_H_23_O_11_	MS^2^ [499]: 163.0392 (100), 191.0555 (33), 173.0448 (9), 179.0340 (7), 337.09310 (6)	Cis-3-pCo, 5CQA
38	7.38	677.17232	677.17145	−1.3	C_31_H_33_O_17_	MS^2^ [677]: 353.0880 (100), 191.0555 (56), 515.1446 (46), 179.0343 (30), 341.0876 (20), 173.0448 (19)	DiCQA-hexoside
39	8.02	515.11950	515.11938	0.98	C_25_H_23_O_12_	MS^2^ [515]: 173.0447 (100), 179.0342 (92), 191.0554 (38), 353.0879 (15), 135.0441 (14), 161.0235 (14)	3,4-DiCQA
40	8.31	515.11950	515.11945	1.05	C_25_H_23_O_12_	MS^2^ [515]: 191.0554 (100), 179.0342 (60), 353.0881 (14), 135.0441 (9)	3,5-DiCQA
41	8.32	335.07724	335.07709	0.95	C_16_H_15_O_8_	MS^2^ [335]: 161.0235 (100), 135.0441 (95), 173.0447 (46), 179.0342 (31)	1-CQL
42	9.12	515.11950	515.11926	0.86	C_25_H_23_O_12_	MS^2^ [515]: 173.0447 (100), 179.0342 (67), 191.0555 (25), 353.0880 (21), 135.0441 (10)	4,5-DiCQA
43	9.22	499.12458	499.12460	1.11	C_25_H_23_O_11_	MS^2^ [499]: 163.0392 (100), 173.0447 (84), 179.0342 (65), 191.0555 (31), 165.0912 (29), 197.0449 (24), 135.0440 (16), 335.0776 (11)	Cis-3-pCo, 5CQA
44	9.54	529.13515	529.13525	0.19	C_26_H_25_O_12_	MS^2^ [529]: 193.0499 (100), 173.0447 (73), 179.0342 (62), 161.0235 (17), 335.0777 (16), 135.0440 (14), 191.0554 (12)	3F, 5CQA
45	9.72	499.12458	499.12515	1.66	C_25_H_23_O_11_	MS^2^ [499]: 191.0555 (100), 163.0392 (39), 179.0343 (33), 173.0447 (13), 135.0441 (8)	3C, 5-pCoQA
46	9.76	529.13515	529.13550	0.66	C_26_H_25_O_12_	MS^2^ [529]: 173.0447 (100), 193.0499 (28), 179.0342 (11)	3F, 5CQA
47	10.11	529.13515	529.13550	0.66	C_26_H_25_O_12_	MS^2^ [529]: 193.0499 (100), 173.0447 (14)	3F, 5CQA
48	10.28	529.13515	529.13550	0.66	C_26_H_25_O_12_	MS^2^ [529]: 191.0554 (100), 193.0499 (61), 179.0341 (33), 173.0447 (19)	3C, 5FQA
49	10.32	677.15119	677.15173	0.79	C_34_H_29_O_15_	MS^2^ [677]: 353.0879 (100), 335.0806 (21), 179.0342 (21), 191.0555 (13)	1,3,5-TriCQA
50	10.75	677.15119	677.15179	0.88	C_34_H_29_O_15_	MS^2^ [677]: 353.0882 (100), 179.0342 (73), 161.0236 (72), 515.1199 (53), 335.0776(29), 173.0448 (27), 497.1096 (13), 191.0556 (8)	1,3,4-TriCQA
51	11.20	497.10893	497.10956	1.72	C_25_H_21_O_11_	MS^2^ [497]: 161.0235 (100), 335.0775 (60), 247.0797 (36), 179.0342 (32), 135.0440 (20)	DiCQL
52	11.26	499.12458	499.12451	1.02	C_25_H_23_O_11_	MS^2^ [499]: 173.0447 (100), 179.0342 (42), 191.0554 (29), 353.0878 (9)	4C, 5-pCoQA
53	11.30	367.10346	367.10199	−3.99	C_17_H_19_O_9_	MS^2^ [367]:173.0447 (100), 134.03625 (35), 191.0499 (15)	4FQA
54	11.62	529.13515	529.13525	0.19	C_26_H_25_O_12_	MS^2^ [529]: 173.0447 (100), 179.0342(27), 191.0555 (24), 193.0500 (11)	4C, 5FQA
55	11.68	497.10893	497.10944	1.60	C_25_H_21_O_11_	MS^2^ [497]:161.0235 (100), 179.0342 (97), 135.0441 (46), 247.0799 (19), 119.0338 (19)	DiCQL
56	12.24	497.10893	497.10950	1.66	C_25_H_21_O_11_	MS^2^ [497]:161.0235 (100), 335.0775 (56), 247.0797 (35), 179.0342 (30), 137.0233 (28), 135.0441 (18)	DiCQL
57	12.27	661.15628	661.15472	−2.36	C_34_H_29_O_14_	MS^2^ [661]: 353.0879 (100), 337.0933 (56), 191.0554 (40), 179.0342 (57), 173.0448 (24), 161.0237 (21), 163.0393 (17)	pCoDiCQA
58	12.28	691.16684	691.16840	2.25	C_35_H_31_O_15_	MS^2^ [691]: 353.0879 (100), 179.0343 (75), 161.0236 (55), 335.0776 (29), 191.0553 (24), 515.1195 (22), 173.0448 (21)	DiCFQA
59	12.38	661.15628	661.15369	−3.92	C_34_H_29_O_14_	MS^2^ [661]: 337.0931 (100), 353.0882 (74), 173.0447 (72), 179.0342 (67), 191.0555 (50), 335.0777 (27), 499.1248 (21), 515.1192 (9)	pCoDiCQA
60	12.56	691.16684	691.16821	1.98	C_35_H_31_O_15_	MS^2^ [691]: 367.1036 (100), 179.0343 (73), 353.0880 (69), 173.0448 (42), 529.1351 (32), 191.0554 (24), 335.0775 (17)	DiCFQA
61	12.59	721.17741	721.17474	−3.70	C_36_H_33_O_16_	MS^2^ [721]: 329.1026 (100), 353.0873 (40), 515.1187 (31), 173.0444 (22), 179.0338 (15), 191.0551 (15), 335.0771 (12), 161.0232 (11), 151.0387 (8)	DiCSQA
62	12.72	677.15119	677.15173	0.79	C_34_H_29_O_15_	MS^2^ [677]: 353.0879 (100), 515.1198 (69), 173.0447 (26), 179.0341 (22), 335.0777 (12), 191.0557 (7), 161.0233 (6)	1,4,5-TriCQA
63	12.77	721.17741	721.17480	−3.62	C_36_H_33_O_16_	MS^2^ [721]: 353.0873 (100), 191.0551 (28), 173.0444 (24), 179.0339 (23), 559.1453 (16), 329.1022 (10), 335.0765 (8), 515.1185 (7), 161.0232 (6)	DiCSQA
64	13.07	721.17741	721.17267	−6.57	C_36_H_33_O_16_	MS^2^ [721]: 353.0873 (100), 173.0443451), 191.0550 (31), 515.1193 (30), 179.0339 (26), 559.1444 (18), 151.0387 (17), 161.0444 (16)	DiCSQA
65	13.11	691.16684	691.16766	1.18	C_35_H_31_O_15_	MS^2^ [691]: 529.1353 (100), 367.1035 (92), 353.0879 (74), 173.0447 (56), 179.0342 (52), 335.0775 (48), 161.0235 (17), 515.1199 (11)	DiCFQA
66	13.28	721.17741	721.17426	−4.37	C_36_H_33_O_16_	MS^2^ [721]: 353.0873 (100), 173.0444 (20), 179.0338 (13), 515.1189 (12), 559.1451 (11), 191.0550 (9), 335.0769 (4)	DiCSQA
67	13.41	499.12458	499.12097	−2.52	C_25_H_23_O_11_	MS^2^ [499]: 191.0554 (100), 173.0447 (46)	3C, 5-pCoQA
68	13.45	721.17741	721.17163	−8.01	C_36_H_33_O_16_	MS^2^ [721]: 353.0874 (100), 173.0443 (25), 559.1452 (19), 179.0338 (14), 191.0552 (13)	DiCSQA

## Data Availability

The data used to support the finding of this study are available from the corresponding author upon request.

## References

[1] Cheung F. (2011). Modern TCM: enter the clinic. *Nature*.

[2] Noetzli M., Ansermot N., Dobrinas M., Eap C. B. (2012). Simultaneous determination of antidementia drugs in human plasma: procedure transfer from HPLC-MS to UPLC-MS/MS. *Journal of Pharmaceutical and Biomedical Analysis*.

[3] Li J., Bai Y., Zhang P. (2017). Simultaneous determination of 5 flavonoids and 7 saponins for quality control of traditional Chinese medicine preparation xinnaoshutong capsule using HPLC-VWD-ELSD. *Journal of Analytical Methods in Chemistry*.

[4] Ren T.-K., Li M.-L., Zheng H., Wang Z., Zhang J.-L. (2020). Characterization of acidic glycosphingolipid changes in C6 glioma rats treated with temozolomide using ultra-high-performance liquid chromatography coupled with quadrupole time-of-flight mass spectrometry. *Journal of Analysis and Testing*.

[5] Jiangsu New Medical College (1977). *Dictionary of Chinese Materia Medica*.

[6] Long N. P., Park S., Anh N. H. (2020). Advances in liquid chromatography-mass spectrometry-based lipidomics: a look ahead. *Journal of Analysis and Testing*.

[7] Seca A. M. L., Grigore A., Pinto D. C. G. A., Silva A. M. S. (2014). The genus inula and their metabolites: from ethnopharmacological to medicinal uses. *Journal of Ethnopharmacology*.

[8] Matuschowski P., Nahrstedt A., Winterhoff H. (2005). Pharmakologische untersuchungen eines frischpflanzenpresssaftes ausCynara scolymusauf choleretische Wirkung. *Zeitschrift für Phytotherapie*.

[9] Cai W., Li K.-L., Xiong P. (2020). A systematic strategy for rapid identification of chlorogenic acids derivatives in Duhaldea nervosa using UHPLC-Q-exactive orbitrap mass spectrometry. *Arabian Journal of Chemistry*.

[10] Masike K., Mhlongo M. I., Mudau S. P. (2017). Highlighting mass spectrometric fragmentation differences and similarities between hydroxycinnamoyl-quinic acids and hydroxycinnamoyl-isocitric acids. *Chemistry Central Journal*.

[11] Ouyang H., Li J., Wu B. (2017). A robust platform based on ultra-high performance liquid chromatography quadrupole time of flight tandem mass spectrometry with a two-step data mining strategy in the investigation, classification, and identification of chlorogenic acids in ainsliaea fragrans champ. *Journal of Chromatography A*.

[12] Jaiswal R., Halabi E. A., Karar M. G. E., Kuhnert N. (2014). Identification and characterisation of the phenolics of Ilex glabra L. gray (aquifoliaceae) leaves by liquid chromatography tandem mass spectrometry. *Phytochemistry*.

[13] Jaiswal R., Matei M. F., Subedi P., Kuhnert N. (2014). Does roasted coffee contain chlorogenic acid lactones or/and cinnamoylshikimate esters?. *Food Research International*.

[14] Jaiswal R., Müller H., Müller A., Karar M. G. E., Kuhnert N. (2014). Identification and characterization of chlorogenic acids, chlorogenic acid glycosides and flavonoids from Lonicera henryi L. (caprifoliaceae) leaves by LC-MS. *Phytochemistry*.

[15] Jaiswal R., Sovdat T., Vivan F., Kuhnert N. (2010). Profiling and characterization by LC-MSn of the chlorogenic acids and hydroxycinnamoylshikimate esters in maté (Ilex paraguariensis). *Journal of Agricultural and Food Chemistry*.

[16] Clifford M. N., Johnston K. L., Knight S., Kuhnert N. (2003). Hierarchical scheme for LC-MSn identification of chlorogenic acids. *Journal of Agricultural and Food Chemistry*.

[17] Clifford M. N., Kirkpatrick J., Kuhnert N., Roozendaal H., Salgado P. R. (2008). LC-MSn analysis of the cis isomers of chlorogenic acids. *Food Chemistry*.

[18] Clifford M. N., Wu W., Kirkpatrick J., Kuhnert N. (2007). Profiling the chlorogenic acids and other caffeic acid derivatives of herbal chrysanthemum by LC−MSn. *Journal of Agricultural and Food Chemistry*.

[19] Clifford M. N., Marks S., Knight S., Kuhnert N. (2006). Characterization by LC-MSn of four new classes of p-coumaric acid-containing diacyl chlorogenic acids in green coffee beans. *Journal of Agricultural and Food Chemistry*.

[20] Liu L., Zhang J., Zheng B. (2018). Rapid characterization of chlorogenic acids in Duhaldea nervosa based on ultra-high-performance liquid chromatography-linear trap quadropole-orbitrap-mass spectrometry and mass spectral trees similarity filter technique. *Journal of Separation Science*.

[21] Clifford M. N., Knight S., Kuhnert N. (2005). Discriminating between the six isomers of dicaffeoylquinic acid by LC-MSn. *Journal of Agricultural and Food Chemistry*.

[22] Gavrilova V., Kajdžanoska M., Gjamovski V., Stefova M. (2011). Separation, characterization and quantification of phenolic compounds in blueberries and red and black currants by HPLC−DAD−ESI-MSn. *Journal of Agricultural and Food Chemistry*.

[23] Zhang J.-Y., Wang Z.-J., Li Y. (2016). A strategy for comprehensive identification of sequential constituents using ultra-high-performance liquid chromatography coupled with linear ion trap-orbitrap mass spectrometer, application study on chlorogenic acids in *Flos lonicerae* japonicae. *Talanta*.

